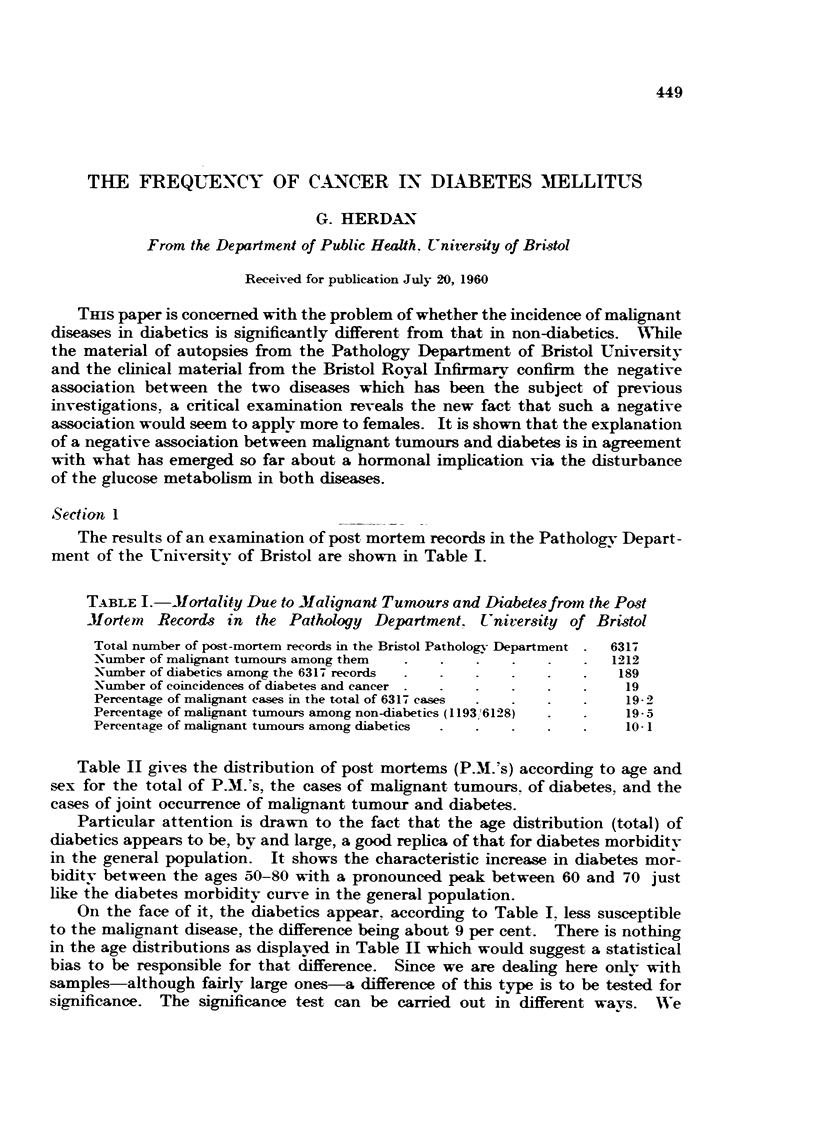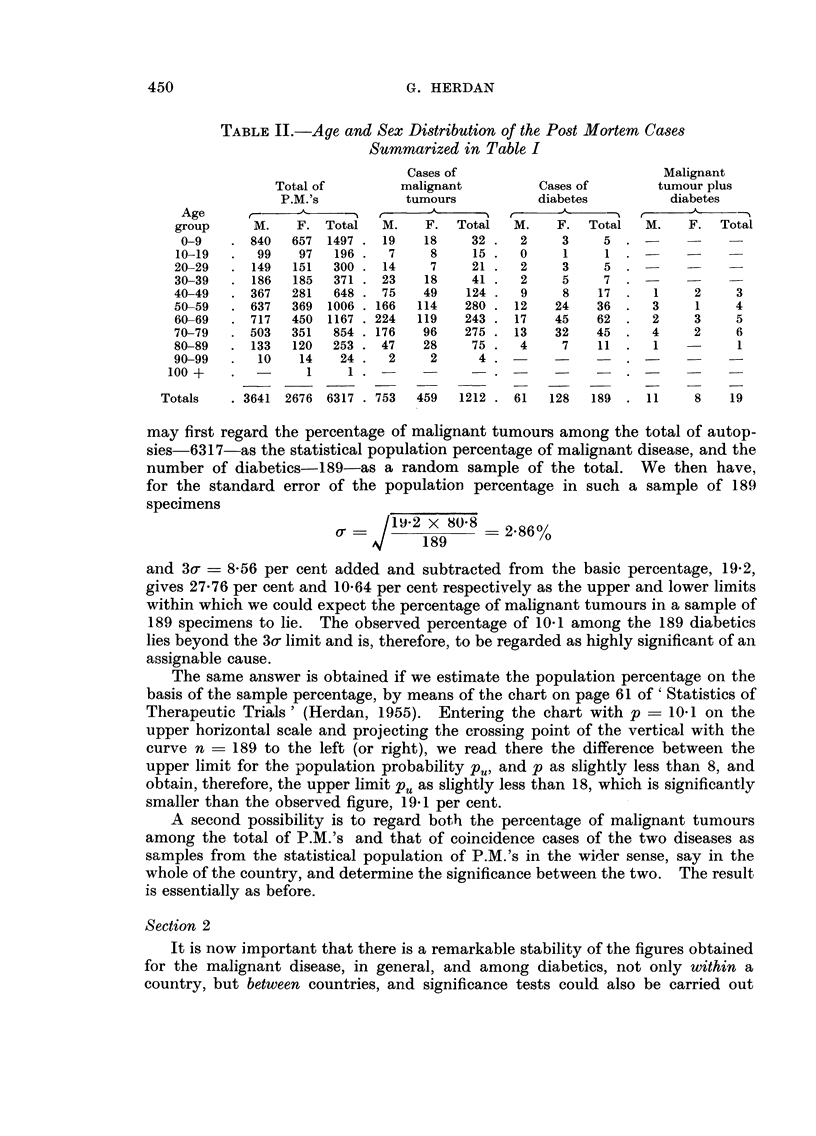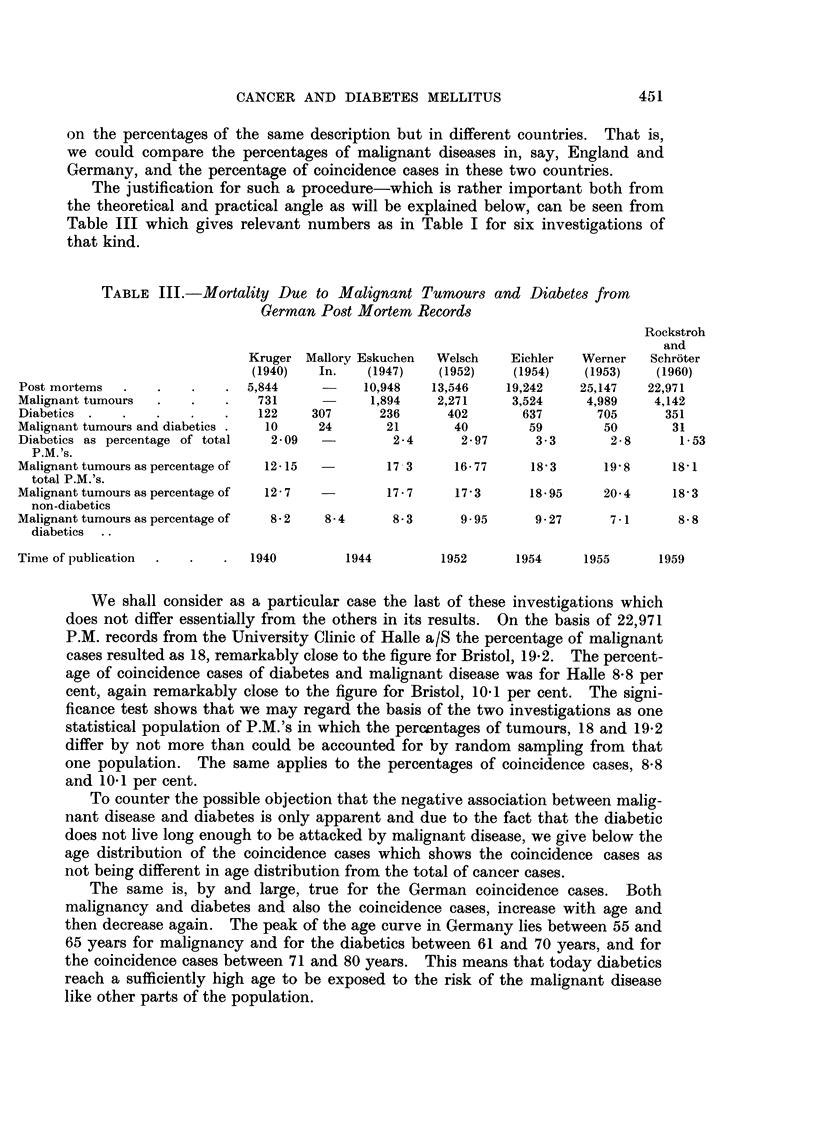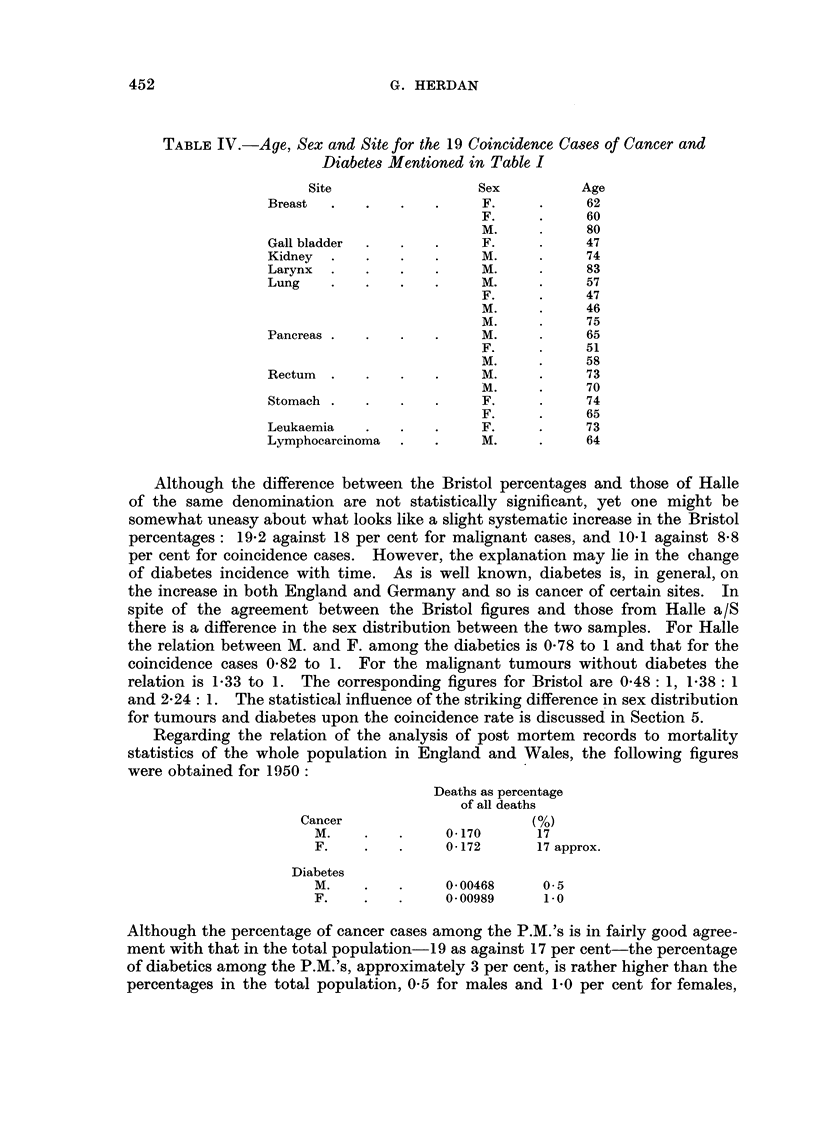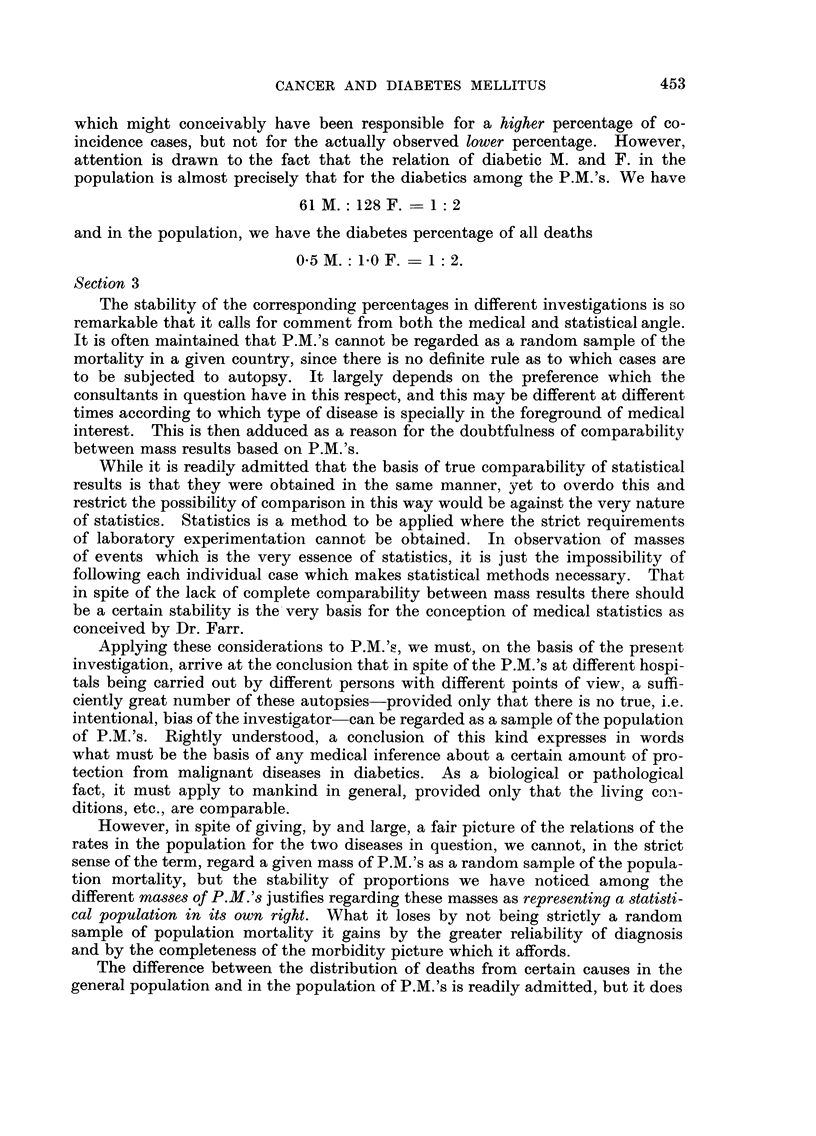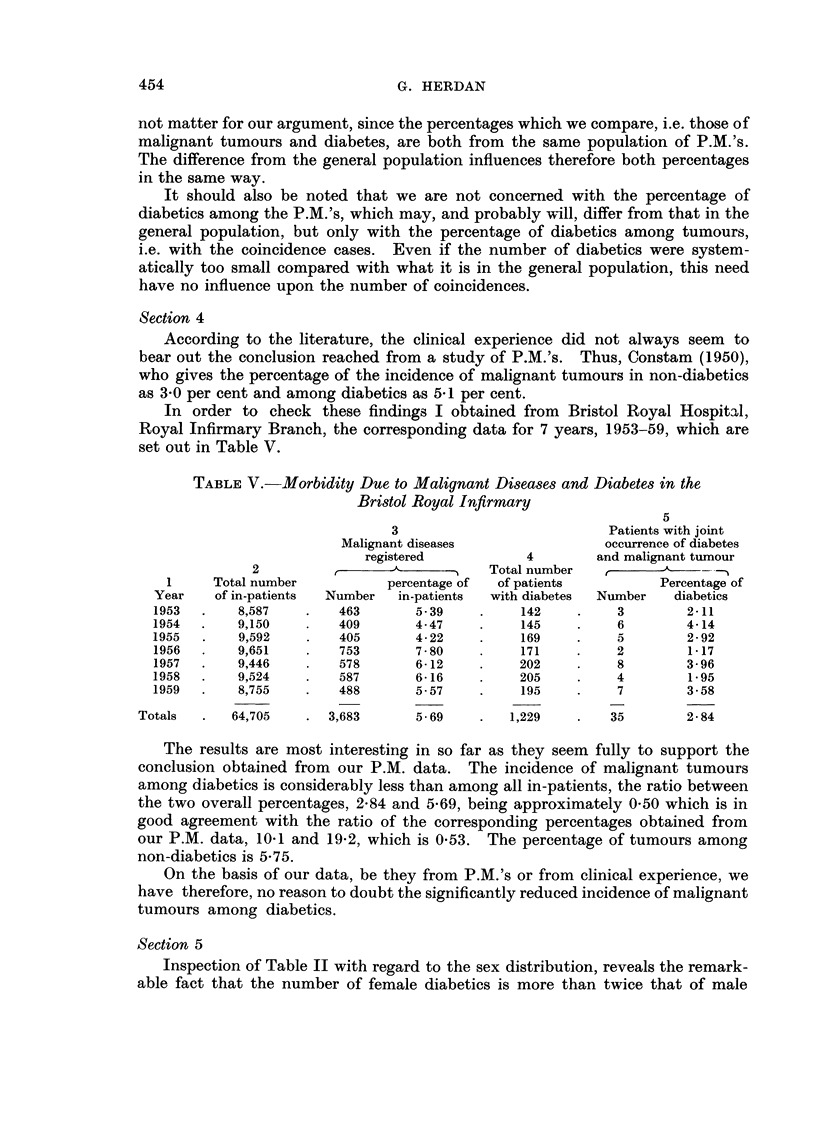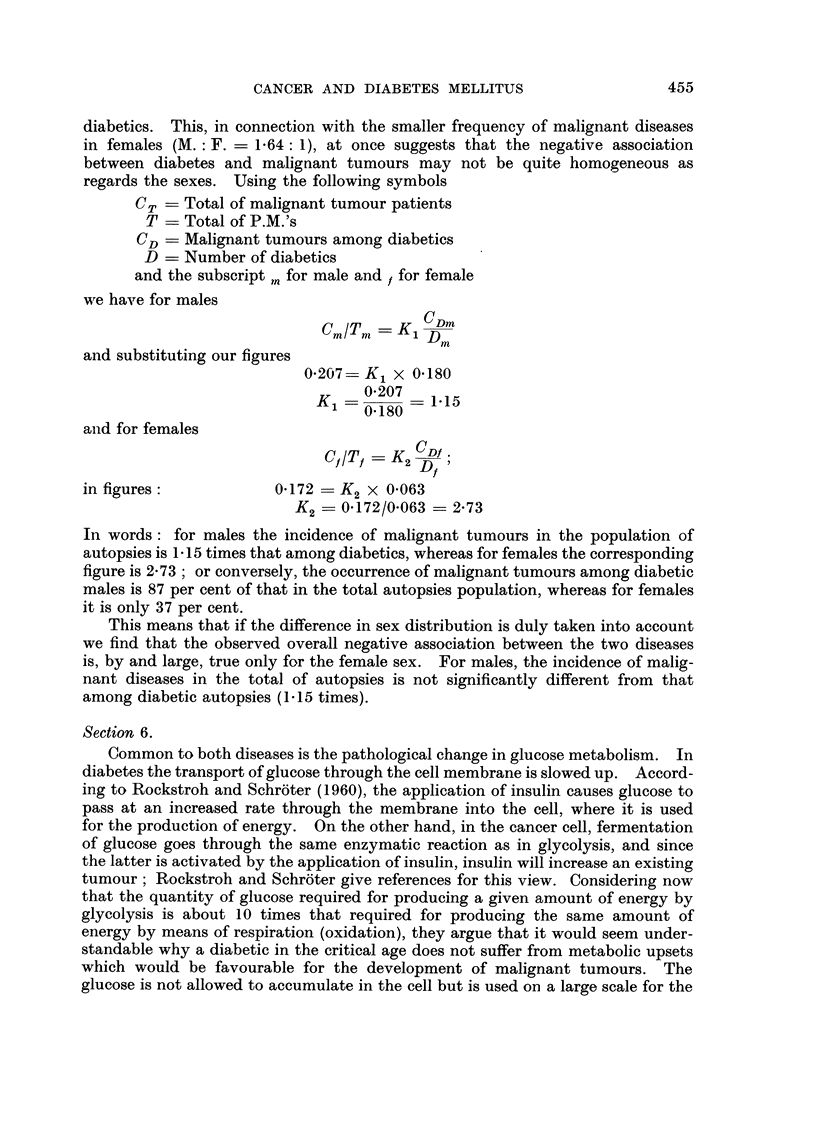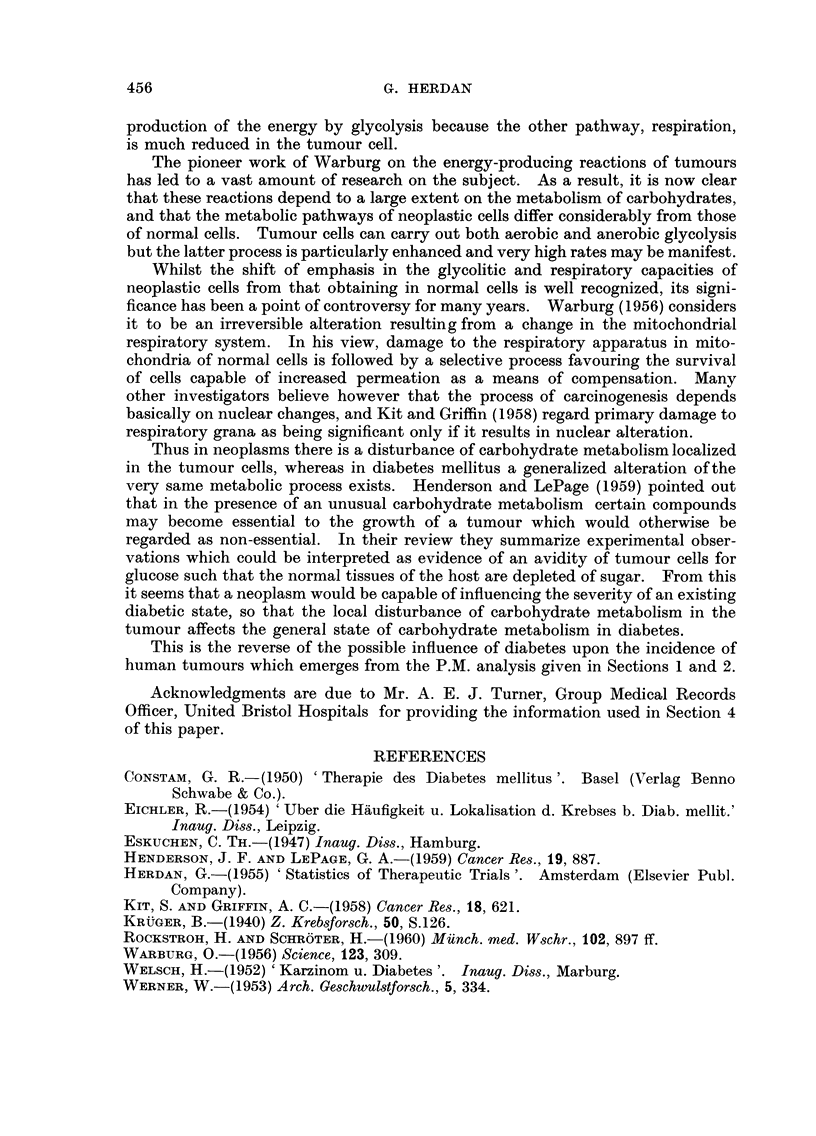# The Frequency of Cancer in Diabetes Mellitus

**DOI:** 10.1038/bjc.1960.48

**Published:** 1960-09

**Authors:** G. Herdan


### THE FREQUENCY OF CANCER IN DIABETES MELLITUS

G. HERDAN

*From the Department of Public Health, University of Bristol*

Received for publication July 20, 1960

THIS paper is concerned with the problem of whether the incidence of malignant diseases in diabetics is significantly different from that in non-diabetics. While the material of autopsies from the Pathology Department of Bristol University and the clinical material from the Bristol Royal Infirmary confirm the negative association between the two diseases which has been the subject of previous investigations, a critical examination reveals the new fact that such a negative association would seem to apply more to females. It is shown that the explanation of a negative association between malignant tumours and diabetes is in agreement with what has emerged so far about a hormonal implication via the disturbance of the glucose metabolism in both diseases.

*Section* 1

The results of an examination of post mortem records in the Pathology Department of the University of Bristol are shown in Table I.

TABLE I.—*Mortality Due to Malignant Tumours and Diabetes from the Post Mortem Records in the Pathology Department, University of Bristol*

| | |
|---|---|
| Total number of post-mortem records in the Bristol Pathology Department . | 6317 |
| Number of malignant tumours among them . . . . . . . | 1212 |
| Number of diabetics among the 6317 records . . . . . . . | 189 |
| Number of coincidences of diabetes and cancer . . . . . . . | 19 |
| Percentage of malignant cases in the total of 6317 cases . . . . . | 19·2 |
| Percentage of malignant tumours among non-diabetics (1193/6128) . . | 19·5 |
| Percentage of malignant tumours among diabetics . . . . . . | 10·1 |

Table II gives the distribution of post mortems (P.M.'s) according to age and sex for the total of P.M.'s, the cases of malignant tumours, of diabetes, and the cases of joint occurrence of malignant tumour and diabetes.

Particular attention is drawn to the fact that the age distribution (total) of diabetics appears to be, by and large, a good replica of that for diabetes morbidity in the general population. It shows the characteristic increase in diabetes morbidity between the ages 50–80 with a pronounced peak between 60 and 70 just like the diabetes morbidity curve in the general population.

On the face of it, the diabetics appear, according to Table I, less susceptible to the malignant disease, the difference being about 9 per cent. There is nothing in the age distributions as displayed in Table II which would suggest a statistical bias to be responsible for that difference. Since we are dealing here only with samples—although fairly large ones—a difference of this type is to be tested for significance. The significance test can be carried out in different ways. We

TABLE II.—*Age and Sex Distribution of the Post Mortem Cases Summarized in Table I*

| Age group | Total of P.M.'s | | | Cases of malignant tumours | | | Cases of diabetes | | | Malignant tumour plus diabetes | | |
|---|---|---|---|---|---|---|---|---|---|---|---|---|
| | M. | F. | Total | M. | F. | Total | M. | F. | Total | M. | F. | Total |
| 0–9 | 840 | 657 | 1497 | 19 | 18 | 32 | 2 | 3 | 5 | — | — | — |
| 10–19 | 99 | 97 | 196 | 7 | 8 | 15 | 0 | 1 | 1 | — | — | — |
| 20–29 | 149 | 151 | 300 | 14 | 7 | 21 | 2 | 3 | 5 | — | — | — |
| 30–39 | 186 | 185 | 371 | 23 | 18 | 41 | 2 | 5 | 7 | — | — | — |
| 40–49 | 367 | 281 | 648 | 75 | 49 | 124 | 9 | 8 | 17 | 1 | 2 | 3 |
| 50–59 | 637 | 369 | 1006 | 166 | 114 | 280 | 12 | 24 | 36 | 3 | 1 | 4 |
| 60–69 | 717 | 450 | 1167 | 224 | 119 | 243 | 17 | 45 | 62 | 2 | 3 | 5 |
| 70–79 | 503 | 351 | 854 | 176 | 96 | 275 | 13 | 32 | 45 | 4 | 2 | 6 |
| 80–89 | 133 | 120 | 253 | 47 | 28 | 75 | 4 | 7 | 11 | 1 | — | 1 |
| 90–99 | 10 | 14 | 24 | 2 | 2 | 4 | — | — | — | — | — | — |
| 100 + | — | 1 | 1 | — | — | — | — | — | — | — | — | — |
| Totals | 3641 | 2676 | 6317 | 753 | 459 | 1212 | 61 | 128 | 189 | 11 | 8 | 19 |

may first regard the percentage of malignant tumours among the total of autopsies—6317—as the statistical population percentage of malignant disease, and the number of diabetics—189—as a random sample of the total. We then have, for the standard error of the population percentage in such a sample of 189 specimens

$$\sigma = \sqrt{\frac{19{\cdot}2 \times 80{\cdot}8}{189}} = 2{\cdot}86\%$$

and $3\sigma = 8{\cdot}56$ per cent added and subtracted from the basic percentage, 19·2, gives 27·76 per cent and 10·64 per cent respectively as the upper and lower limits within which we could expect the percentage of malignant tumours in a sample of 189 specimens to lie. The observed percentage of 10·1 among the 189 diabetics lies beyond the $3\sigma$ limit and is, therefore, to be regarded as highly significant of an assignable cause.

The same answer is obtained if we estimate the population percentage on the basis of the sample percentage, by means of the chart on page 61 of ' Statistics of Therapeutic Trials ' (Herdan, 1955). Entering the chart with $p = 10{\cdot}1$ on the upper horizontal scale and projecting the crossing point of the vertical with the curve $n = 189$ to the left (or right), we read there the difference between the upper limit for the population probability $p_u$, and $p$ as slightly less than 8, and obtain, therefore, the upper limit $p_u$ as slightly less than 18, which is significantly smaller than the observed figure, 19·1 per cent.

A second possibility is to regard both the percentage of malignant tumours among the total of P.M.'s and that of coincidence cases of the two diseases as samples from the statistical population of P.M.'s in the wider sense, say in the whole of the country, and determine the significance between the two. The result is essentially as before.

*Section* 2

It is now important that there is a remarkable stability of the figures obtained for the malignant disease, in general, and among diabetics, not only *within* a country, but *between* countries, and significance tests could also be carried out on the percentages of the same description but in different countries. That is, we could compare the percentages of malignant diseases in, say, England and Germany, and the percentage of coincidence cases in these two countries.

The justification for such a procedure—which is rather important both from the theoretical and practical angle as will be explained below, can be seen from Table III which gives relevant numbers as in Table I for six investigations of that kind.

TABLE III.—*Mortality Due to Malignant Tumours and Diabetes from German Post Mortem Records*

| | Kruger (1940) | Mallory In. | Eskuchen (1947) | Welsch (1952) | Eichler (1954) | Werner (1953) | Rockstroh and Schröter (1960) |
|---|---|---|---|---|---|---|---|
| Post mortems . . . . | 5,844 | — | 10,948 | 13,546 | 19,242 | 25,147 | 22,971 |
| Malignant tumours . . . | 731 | — | 1,894 | 2,271 | 3,524 | 4,989 | 4,142 |
| Diabetics . . . . . . | 122 | 307 | 236 | 402 | 637 | 705 | 351 |
| Malignant tumours and diabetics . | 10 | 24 | 21 | 40 | 59 | 50 | 31 |
| Diabetics as percentage of total P.M.'s. | 2·09 | — | 2·4 | 2·97 | 3·3 | 2·8 | 1·53 |
| Malignant tumours as percentage of total P.M.'s. | 12·15 | — | 17 3 | 16·77 | 18·3 | 19·8 | 18·1 |
| Malignant tumours as percentage of non-diabetics | 12·7 | — | 17·7 | 17·3 | 18·95 | 20·4 | 18·3 |
| Malignant tumours as percentage of diabetics . . | 8·2 | 8·4 | 8·3 | 9·95 | 9·27 | 7·1 | 8·8 |
| Time of publication . . . | 1940 | 1944 | | 1952 | 1954 | 1955 | 1959 |

We shall consider as a particular case the last of these investigations which does not differ essentially from the others in its results. On the basis of 22,971 P.M. records from the University Clinic of Halle a/S the percentage of malignant cases resulted as 18, remarkably close to the figure for Bristol, 19·2. The percentage of coincidence cases of diabetes and malignant disease was for Halle 8·8 per cent, again remarkably close to the figure for Bristol, 10·1 per cent. The significance test shows that we may regard the basis of the two investigations as one statistical population of P.M.'s in which the percentages of tumours, 18 and 19·2 differ by not more than could be accounted for by random sampling from that one population. The same applies to the percentages of coincidence cases, 8·8 and 10·1 per cent.

To counter the possible objection that the negative association between malignant disease and diabetes is only apparent and due to the fact that the diabetic does not live long enough to be attacked by malignant disease, we give below the age distribution of the coincidence cases which shows the coincidence cases as not being different in age distribution from the total of cancer cases.

The same is, by and large, true for the German coincidence cases. Both malignancy and diabetes and also the coincidence cases, increase with age and then decrease again. The peak of the age curve in Germany lies between 55 and 65 years for malignancy and for the diabetics between 61 and 70 years, and for the coincidence cases between 71 and 80 years. This means that today diabetics reach a sufficiently high age to be exposed to the risk of the malignant disease like other parts of the population.

TABLE IV.—*Age, Sex and Site for the* 19 *Coincidence Cases of Cancer and Diabetes Mentioned in Table I*

| Site | Sex | Age |
|---|---|---|
| Breast | F. | 62 |
| | F. | 60 |
| | M. | 80 |
| Gall bladder | F. | 47 |
| Kidney | M. | 74 |
| Larynx | M. | 83 |
| Lung | M. | 57 |
| | F. | 47 |
| | M. | 46 |
| | M. | 75 |
| Pancreas | M. | 65 |
| | F. | 51 |
| | M. | 58 |
| Rectum | M. | 73 |
| | M. | 70 |
| Stomach | F. | 74 |
| | F. | 65 |
| Leukaemia | F. | 73 |
| Lymphocarcinoma | M. | 64 |

Although the difference between the Bristol percentages and those of Halle of the same denomination are not statistically significant, yet one might be somewhat uneasy about what looks like a slight systematic increase in the Bristol percentages : 19·2 against 18 per cent for malignant cases, and 10·1 against 8·8 per cent for coincidence cases. However, the explanation may lie in the change of diabetes incidence with time. As is well known, diabetes is, in general, on the increase in both England and Germany and so is cancer of certain sites. In spite of the agreement between the Bristol figures and those from Halle a/S there is a difference in the sex distribution between the two samples. For Halle the relation between M. and F. among the diabetics is 0·78 to 1 and that for the coincidence cases 0·82 to 1. For the malignant tumours without diabetes the relation is 1·33 to 1. The corresponding figures for Bristol are 0·48 : 1, 1·38 : 1 and 2·24 : 1. The statistical influence of the striking difference in sex distribution for tumours and diabetes upon the coincidence rate is discussed in Section 5.

Regarding the relation of the analysis of post mortem records to mortality statistics of the whole population in England and Wales, the following figures were obtained for 1950 :

| | Deaths as percentage of all deaths | (%) |
|---|---|---|
| Cancer | | |
| M. | 0·170 | 17 |
| F. | 0·172 | 17 approx. |
| Diabetes | | |
| M. | 0·00468 | 0·5 |
| F. | 0·00989 | 1·0 |

Although the percentage of cancer cases among the P.M.'s is in fairly good agreement with that in the total population—19 as against 17 per cent—the percentage of diabetics among the P.M.'s, approximately 3 per cent, is rather higher than the percentages in the total population, 0·5 for males and 1·0 per cent for females, which might conceivably have been responsible for a *higher* percentage of coincidence cases, but not for the actually observed *lower* percentage. However, attention is drawn to the fact that the relation of diabetic M. and F. in the population is almost precisely that for the diabetics among the P.M.'s. We have

61 M. : 128 F. = 1 : 2

and in the population, we have the diabetes percentage of all deaths

0·5 M. : 1·0 F. = 1 : 2.

*Section* 3

The stability of the corresponding percentages in different investigations is so remarkable that it calls for comment from both the medical and statistical angle. It is often maintained that P.M.'s cannot be regarded as a random sample of the mortality in a given country, since there is no definite rule as to which cases are to be subjected to autopsy. It largely depends on the preference which the consultants in question have in this respect, and this may be different at different times according to which type of disease is specially in the foreground of medical interest. This is then adduced as a reason for the doubtfulness of comparability between mass results based on P.M.'s.

While it is readily admitted that the basis of true comparability of statistical results is that they were obtained in the same manner, yet to overdo this and restrict the possibility of comparison in this way would be against the very nature of statistics. Statistics is a method to be applied where the strict requirements of laboratory experimentation cannot be obtained. In observation of masses of events which is the very essence of statistics, it is just the impossibility of following each individual case which makes statistical methods necessary. That in spite of the lack of complete comparability between mass results there should be a certain stability is the very basis for the conception of medical statistics as conceived by Dr. Farr.

Applying these considerations to P.M.'s, we must, on the basis of the present investigation, arrive at the conclusion that in spite of the P.M.'s at different hospitals being carried out by different persons with different points of view, a sufficiently great number of these autopsies—provided only that there is no true, i.e. intentional, bias of the investigator—can be regarded as a sample of the population of P.M.'s. Rightly understood, a conclusion of this kind expresses in words what must be the basis of any medical inference about a certain amount of protection from malignant diseases in diabetics. As a biological or pathological fact, it must apply to mankind in general, provided only that the living conditions, etc., are comparable.

However, in spite of giving, by and large, a fair picture of the relations of the rates in the population for the two diseases in question, we cannot, in the strict sense of the term, regard a given mass of P.M.'s as a random sample of the population mortality, but the stability of proportions we have noticed among the different *masses of P.M.'s* justifies regarding these masses as *representing a statistical population in its own right.* What it loses by not being strictly a random sample of population mortality it gains by the greater reliability of diagnosis and by the completeness of the morbidity picture which it affords.

The difference between the distribution of deaths from certain causes in the general population and in the population of P.M.'s is readily admitted, but it does not matter for our argument, since the percentages which we compare, i.e. those of malignant tumours and diabetes, are both from the same population of P.M.'s. The difference from the general population influences therefore both percentages in the same way.

It should also be noted that we are not concerned with the percentage of diabetics among the P.M.'s, which may, and probably will, differ from that in the general population, but only with the percentage of diabetics among tumours, i.e. with the coincidence cases. Even if the number of diabetics were systematically too small compared with what it is in the general population, this need have no influence upon the number of coincidences.

*Section* 4

According to the literature, the clinical experience did not always seem to bear out the conclusion reached from a study of P.M.'s. Thus, Constam (1950), who gives the percentage of the incidence of malignant tumours in non-diabetics as 3·0 per cent and among diabetics as 5·1 per cent.

In order to check these findings I obtained from Bristol Royal Hospital, Royal Infirmary Branch, the corresponding data for 7 years, 1953–59, which are set out in Table V.

TABLE V.—*Morbidity Due to Malignant Diseases and Diabetes in the Bristol Royal Infirmary*

| 1 | 2 | 3 Malignant diseases registered | | 4 | 5 Patients with joint occurrence of diabetes and malignant tumour | |
|---|---|---|---|---|---|---|
| Year | Total number of in-patients | Number | percentage of in-patients | Total number of patients with diabetes | Number | Percentage of diabetics |
| 1953 | 8,587 | 463 | 5·39 | 142 | 3 | 2·11 |
| 1954 | 9,150 | 409 | 4·47 | 145 | 6 | 4·14 |
| 1955 | 9,592 | 405 | 4·22 | 169 | 5 | 2·92 |
| 1956 | 9,651 | 753 | 7·80 | 171 | 2 | 1·17 |
| 1957 | 9,446 | 578 | 6·12 | 202 | 8 | 3·96 |
| 1958 | 9,524 | 587 | 6·16 | 205 | 4 | 1·95 |
| 1959 | 8,755 | 488 | 5·57 | 195 | 7 | 3·58 |
| Totals | 64,705 | 3,683 | 5·69 | 1,229 | 35 | 2·84 |

The results are most interesting in so far as they seem fully to support the conclusion obtained from our P.M. data. The incidence of malignant tumours among diabetics is considerably less than among all in-patients, the ratio between the two overall percentages, 2·84 and 5·69, being approximately 0·50 which is in good agreement with the ratio of the corresponding percentages obtained from our P.M. data, 10·1 and 19·2, which is 0·53. The percentage of tumours among non-diabetics is 5·75.

On the basis of our data, be they from P.M.'s or from clinical experience, we have therefore, no reason to doubt the significantly reduced incidence of malignant tumours among diabetics.

*Section* 5

Inspection of Table II with regard to the sex distribution, reveals the remarkable fact that the number of female diabetics is more than twice that of male diabetics. This, in connection with the smaller frequency of malignant diseases in females (M. : F. = 1·64 : 1), at once suggests that the negative association between diabetes and malignant tumours may not be quite homogeneous as regards the sexes. Using the following symbols

$C_T$ = Total of malignant tumour patients
$T$ = Total of P.M.'s
$C_D$ = Malignant tumours among diabetics
$D$ = Number of diabetics
and the subscript $_m$ for male and $_f$ for female we have for males

$$C_m/T_m = K_1 \frac{C_{Dm}}{D_m}$$

and substituting our figures

$$0{\cdot}207 = K_1 \times 0{\cdot}180$$
$$K_1 = \frac{0{\cdot}207}{0{\cdot}180} = 1{\cdot}15$$

and for females

$$C_f/T_f = K_2 \frac{C_{Df}}{D_f}\,;$$

in figures :

$$0{\cdot}172 = K_2 \times 0{\cdot}063$$

$$K_2 = 0{\cdot}172/0{\cdot}063 = 2{\cdot}73$$

In words : for males the incidence of malignant tumours in the population of autopsies is 1·15 times that among diabetics, whereas for females the corresponding figure is 2·73 ; or conversely, the occurrence of malignant tumours among diabetic males is 87 per cent of that in the total autopsies population, whereas for females it is only 37 per cent.

This means that if the difference in sex distribution is duly taken into account we find that the observed overall negative association between the two diseases is, by and large, true only for the female sex. For males, the incidence of malignant diseases in the total of autopsies is not significantly different from that among diabetic autopsies (1·15 times).

*Section* 6.

Common to both diseases is the pathological change in glucose metabolism. In diabetes the transport of glucose through the cell membrane is slowed up. According to Rockstroh and Schröter (1960), the application of insulin causes glucose to pass at an increased rate through the membrane into the cell, where it is used for the production of energy. On the other hand, in the cancer cell, fermentation of glucose goes through the same enzymatic reaction as in glycolysis, and since the latter is activated by the application of insulin, insulin will increase an existing tumour ; Rockstroh and Schröter give references for this view. Considering now that the quantity of glucose required for producing a given amount of energy by glycolysis is about 10 times that required for producing the same amount of energy by means of respiration (oxidation), they argue that it would seem understandable why a diabetic in the critical age does not suffer from metabolic upsets which would be favourable for the development of malignant tumours. The glucose is not allowed to accumulate in the cell but is used on a large scale for the production of the energy by glycolysis because the other pathway, respiration, is much reduced in the tumour cell.

The pioneer work of Warburg on the energy-producing reactions of tumours has led to a vast amount of research on the subject. As a result, it is now clear that these reactions depend to a large extent on the metabolism of carbohydrates, and that the metabolic pathways of neoplastic cells differ considerably from those of normal cells. Tumour cells can carry out both aerobic and anerobic glycolysis but the latter process is particularly enhanced and very high rates may be manifest.

Whilst the shift of emphasis in the glycolitic and respiratory capacities of neoplastic cells from that obtaining in normal cells is well recognized, its significance has been a point of controversy for many years. Warburg (1956) considers it to be an irreversible alteration resulting from a change in the mitochondrial respiratory system. In his view, damage to the respiratory apparatus in mitochondria of normal cells is followed by a selective process favouring the survival of cells capable of increased permeation as a means of compensation. Many other investigators believe however that the process of carcinogenesis depends basically on nuclear changes, and Kit and Griffin (1958) regard primary damage to respiratory grana as being significant only if it results in nuclear alteration.

Thus in neoplasms there is a disturbance of carbohydrate metabolism localized in the tumour cells, whereas in diabetes mellitus a generalized alteration of the very same metabolic process exists. Henderson and LePage (1959) pointed out that in the presence of an unusual carbohydrate metabolism certain compounds may become essential to the growth of a tumour which would otherwise be regarded as non-essential. In their review they summarize experimental observations which could be interpreted as evidence of an avidity of tumour cells for glucose such that the normal tissues of the host are depleted of sugar. From this it seems that a neoplasm would be capable of influencing the severity of an existing diabetic state, so that the local disturbance of carbohydrate metabolism in the tumour affects the general state of carbohydrate metabolism in diabetes.

This is the reverse of the possible influence of diabetes upon the incidence of human tumours which emerges from the P.M. analysis given in Sections 1 and 2.

Acknowledgments are due to Mr. A. E. J. Turner, Group Medical Records Officer, United Bristol Hospitals for providing the information used in Section 4 of this paper.

### REFERENCES

Constam, G. R.—(1950) ‘Therapie des Diabetes mellitus’. Basel (Verlag Benno Schwabe & Co.).
Eichler, R.—(1954) ‘Uber die Häufigkeit u. Lokalisation d. Krebses b. Diab. mellit.’ *Inaug. Diss.*, Leipzig.
Eskuchen, C. Th.—(1947) *Inaug. Diss.*, Hamburg.
Henderson, J. F. and LePage, G. A.—(1959) *Cancer Res.*, **19**, 887.
Herdan, G.—(1955) ‘Statistics of Therapeutic Trials’. Amsterdam (Elsevier Publ. Company).
Kit, S. and Griffin, A. C.—(1958) *Cancer Res.*, **18**, 621.
Krüger, B.—(1940) *Z. Krebsforsch.*, **50**, S.126.
Rockstroh, H. and Schröter, H.—(1960) *Münch. med. Wschr.*, **102**, 897 ff.
Warburg, O.—(1956) *Science*, **123**, 309.
Welsch, H.—(1952) ‘Karzinom u. Diabetes’. *Inaug. Diss.*, Marburg.
Werner, W.—(1953) *Arch. Geschwulstforsch.*, **5**, 334.